# A Review on Biomaterials in Dental Implantology

**Published:** 2015-09

**Authors:** Hariprasad Ananth, Vinaya Kundapur, H. S. Mohammed, M. Anand, G. S. Amarnath, Sunil Mankar

**Affiliations:** 1 Department of Prosthodontics, MR Ambedkar Dental College, Bangalore, India;; 2 Department of Endodontics, DA Pandu Memorial RV Dental College, Bangalore, India

**Keywords:** Titanium, Osteointegration, Zirconia, Sandblasting, Bioglass, Hydroxyapetite

## Abstract

Implants have been gaining popularity amongst the patients and frequently are being considered as a first treatment option. Modern dentistry is beginning to understand, realize, and utilize the benefits of biotechnology in health care. Study of material sciences along with the biomechanical sciences provides optimization of design and material concepts for surgical implants. Biocompatibility is property of implant material to show favorable response in given biological environment. In attempt to replace a missing tooth many biomaterials have been evolved as implants for many years in an effort to create an optimal interaction between the body and the implanted material. With all the advancements and developments in the science and technology, the materials available for dental implants also improved. The choice of material for a particular implant application will generally be a compromise to meet many different required properties. There is, however, one aspect that is always of prime importance that how the tissue at the implant site responds to the biochemical disturbance that a foreign material presents.

## INTRODUCTION

The development and modification of dental implants have taken place for many years in an effort to create an optimal interaction between the body and the implanted material. The goal of achieving an optimal bone-implant interface has been approached by the alteration of implant surface topography, chemistry, energy and charge as well as bulk material composition. Schmidt *et al*. *(2001) defines an ideal bone implant* material as having a biocompatible chemical composition to avoid adverse tissue reaction, excellent corrosion resistance in the physiologic limits, acceptable strength, a high resistance to wear and a modulus of elasticity similar to that of bone to minimize bone resorption around the implant (1).

The choice of material for a particular implant application will generally be a compromise to meet many different required properties. There is, however, one aspect that is always of prime importance that how the tissue at the implant site responds to the biochemical disturbance that a foreign material presents. Dr. Jonathan Black suggested that the term “biologic performance” is more appropriate than biocompatibility to represent the various interactions between host and the material (2).

Biocompatibility is dependent on the basic bulk and surface properties of the biomaterial. All aspects of basic manufacturing, finishing, packaging and delivering, sterilizing, and placing (including surgical) must be adequately controlled to ensure clean and non traumatizing conditions.

## HISTORIC BACKGROUND

Implant designs are traceable to early Egyptians and South Central American cultures and have evolved into the present implant designs that are now experiencing explosive popularity. The earliest dental implants were of stone and ivory cited in archeological records of China and Egypt before the Common Era. Gold and Ivory dental implant were used in the 16th & 17th centuries. Metal implant devices of Gold, Lead Iridium, Tantalum, Stainless Steel and Cobalt alloys were developed in the early 20th century (3).

Ancient Egyptians in 2500 B.C. attempted to stabilize periodontally compromised teeth with the use of gold ligature wire. Implanted animal teeth carved of ivory cited in ancient Egyptian writings are the oldest examples of primitive implantology. Dating to approximately 500 B.C. the Etruscan population utilized soldered golden bands incorporating animal teeth to restore masticatory efficiency. The Phoenician population in the same era utilized gold wire to stabilize periodontally compromised teeth (4).

Sea shells were used in place of teeth in 600 AD evidence of which was found in Honduras, & tooth restorations made of Jade & Turquoise, were found in Mayan skulls. Albucasis de Condue (936-1013) of france used ox bone to replace missing teeth; this was one of the early documented placement of implant. In 1700’s John Hunter suggested the possibility of transplanting teeth of one human to another. Towards the 18th century, Pierre Fauchard and John Hunter further documented tooth transportation with conditions for its success. They claimed that success was greater with anterior teeth or premolar replacement and in young people with healthy tooth sockets (5).

Titanium Sometimes called the “space age metal”. It was discovered in england by William Gregor in 1791 & Named by Martin Heinrich Klaproth for Titans of greek mythology in 1795. Impure titanium was prepared by Nilson and Pettersson in 1887 (6).

Zirconium dioxide (Zro_2_) was accidentally identified by the German chemist Martin Heinrich Klaproth in 1789 (7).

In 1809, Maggiolo fabricated gold roots that were fixed to teeth by means of a spring. These single, tooth size gold implant were placed without a crown to heal passively in a fresh extraction site just above the gingival (that were not truly submerged into bone). The crown was added after healing. The insertion of such roots of gold was in exitable followed by intense pain and gingival inflammation (5).

Harris followed in 1887 with the implantation of a platinum post coated with lead. The post was shaped like a tooth root and the lead was roughened for retention in the socket (5). Bonwell in 1895 used gold and iridium tubes implanted into bone to restore a single tooth as to support complete dentures. Payne, in 1898 implanted a silver capsule as a foundation for a porcelain crown that was cemented several weeks later. Scholl in 1905 demonstrated a porcelain corrugated root implant. The implant was successful for two years and was anchored to adjacent teeth and fillings through the use of pins (5).

Pure metallic titanium (99.9%) was first prepared in 1910 by Matthew A. Hunter by heating TiCl_4_ with sodium in a steel bomb at 700–800°C in the Hunter process.In1932 William Justin Kroll produced Titanium by reducing titanium tetrachloride (TiCl_4_) with calcium. Eight years later he refined this process by using magnesium and even sodium in what became known as the Kroll process (8).

Greenfields in 1913 introduced & patented hollow ‘basket’ implant made of mesh work of 24 gauge iridium-platinum wires soldered with 24 karat gold. This was used to support single implant as well as fixed dental prosthesis comprising as many as eight implants (5).

After 1925 modern era started with advancement in implant biomaterials. In 1937 Venable et al analyzed the interactions of cobalt alloy & other available metals and alloys with bone. They concluded that certain metals produced a galvanic reaction, which led to corrosion when these metals contacted tissue fluids. Strock in 1939 described a method of placing a vitallium screw to provide anchorage for placement of a missing tooth. Vitallium, a cast alloy composed of cobalt, chromium & molybdenum was considered to be relatively inert, compactible with living tissues and resistant to adverse reaction with body fluids. Early evaluations documented vitallium implant with survival rates of 10 or more years. Vitallium has also been used in different forms of surgical devices, such as dental subperiosteal implant & orthopedic plates, screws, nails & joints (5).

Formiggni in 1947 developed a single helix wire spiral implant made from tantalum or stainless steel. At the same time, Raphael Chercheve designed a double delinked spiral implants made of a chrome-cobalt alloy. In 1948 Goldberg & Gershkoff reported insertion of first viable subperiosteal implant. In 1952 Branemark developed a threaded implant design made of pure titanium that increased the popularity of implants to new levels. Branemark studied every aspect of implant design, including biological, mechanical, physiological & functional phenomena relative to success of endosteal implant. A very important aspect of this evolution-which including biomaterials, designs & clinical application-was the clinical research led by P I Branemark in Sweden. His studies utilized unalloyed titanium, a root form design & very controlled conditions for surgery, restoration and maintenance. In 1963 Linkow designed & introduced the hollow basket design with with vents & screws threads (9).

There are four types of implant designs that have evolved during the centuries of development. The first and most common type is ENDOSTEAL implants placed into alveolar /basal bone of mandible or maxilla & transects only one cortical plate. One such example is Blade Implant developed in 1967 by two groups led by Linkow & Roberts. Other example being Ramus Frame Implant which is horse shoe shaped stainless steel device inserted into the mandible from one retromolar pad to other, passing through anterior symphysis area. The most popular endosteal implant is Root Form Implant designed to mimic the shape of the tooth roots for directional load distribution and proper positioning in bone (9).

The second implant design is SUBPERIOSTEAL implant which employs an implant substructure & superstructure where the custom cast frame is placed directly beneath the periosteum overlying bony cortex this was developed by Dhal (1940) & refined by Berman (1951) who used a direct bone impression technique. The third type of design is TRANSOSTEAL implant combines the subperiosteal & endosteal components. This type penetrates both cortical plates & passes through full thickness of alveolar bone. The concept of transosseous implants was first conceived in germany in early 1930’s Small (1968) developed the mandibular staple implant, which was modified by Bosker (1982) with the transmandibular implant made of gold alloy. The fourth type is EPITHELIAL implant, which is inserted into the oral mucosa (9). Irrespective of implant design failure can occur due to local and systemic factors during replacement of missing teeth in surgical implant placement procedure.

Modern implants come in a variety of shapes and sizes to suit the different teeth they replace, and for the types of prosthetic (false) teeth they will replace. Their surfaces have been improved to enhance the osseointegration process. Instead of being smooth or machined, they are generally roughened by sandblasting and acid etching, which dramatically increases the surface area to which bone can attach.

## TYPES OF BIOMATERIALS

### Dental Implant Materials

Metals and Alloys (Titanium & Titanium –6 Aluminum-4Vanadium (Ti-6AI- 4V) and cp Ti, Cobalt-Chromium-Molybdenum-Based Alloy, Iron-Chromium-Nickel-Based Alloys), Ceramics (Aluminum, Titanium and Zirconium oxide, Bioactive and biodegradable ceramics) Carbon Carbon & carbon silicon, Vitreous and Pyrolytic) Polymers and Composites (Polymethylmethacrylate (PMMA), Polyethylene (UHMW-PE), Polytetrafluoroethylene (PTFE), Silicone rubber, Polysulfone) (10).

### Bone Augmentation Materials

CERAMICS (Calcium phosphate, Bioactive glass & glass ceramics), POLYMERS (PMMA, Lactic/glycolic acid), NATURAL MINERALS (Collagen, Demineralized bone matrix, Bone morphogenic proteins) (10).

## TITANIUM

Titanium exists in nature as a pure element with an atomic number 22, with atomic weight 47.9.Titanium makes up about 0.6% of earth’s crust and is a million times more abundant than gold. This metal exists as Rutile (TiO_2_) or Ilmenite (FeTiO_3_) compounds and requires specific extraction methods to be recovered in its elemental state. The Hunter process involves reduction of TiCl_4_ with sodium & Kroll process involves reduction of TiCl_4_ by calcium, magnesium, where as Iodide process involves formation of titanium iodide through the reaction of titanium with iodide. The titanium iodide is later decomposed on a heated titanium wire (5). Titanium & its alloys are used in dentistry as prosthetic appliances because of its unique combination of chemical, physical & biological properties. ASTM International (the American Society for Testing and Materials) recognizes four grades of commercially pure titanium, or Ti, and three titanium alloys (Ti-6Al-4V, Ti-6Al-4V Extra Low Interstitial [low components] and Ti-Al-Nb) (6). Clinically two forms of titanium have received the most interest, one is the commercially pure titanium (Ti-160) & the other alloy of Ti-6%Al-4% Va (Ti-318) [N.B all alloys are given in wt %] (11).

### Commercially Pure Titanium (CPTi)

Commercially pure titanium is an alloy of titanium & oxygen. To satisfy the British standard specification for use in surgical implants the oxygen content must be less than 0.5% (12). In this form the alloy has a close packed hexagonal structure. The oxygen is in solution so that metal is single phase. Elements such as oxygen, nitrogen & carbon have a greater solubility in the close packed hexagonal structure of the alpha phase than in cubic form of beta phase. These elements form interstitial solid solutions with titanium & help to stabilise the alpha phase. Transition elements such as molybdenum, niobium& vanadium act as beta stabilisers (11).

### Ti-6%Al-4%V

Titanium undergoes a transformation from a hexagonal close packed alpha phase to a body centered cubic beta phase at 883°C, alloying elements can be added to stabilize either phase. When aluminium & vanadium are added to titanium in small quantities the strength of alloy is much increased over that of commercially pure titanium. Aluminium is considered to be an alpha stabiliser & with vanadium acting as beta stabiliser, which is used to minimize the formation of TiAl_3_ to approximately 6% or less to decrease the alloy’s susceptibility to corrosion (5). The temperature at which the alpha beta transition occurs is depressed such that both alpha & beta forms can exist at room temperature (13). Ti-6%Al-4%V has a two phase structure of alpha & beta grains. In situations where extra hardness is needed Ti-550, An alloy of Ti-4%Mo-4%Al-2% Sn, is being used instead of Ti -318. A new wrought Ti 6% Al 7% Nb has been a great promise as an implant material (11).

### Properties of titanium

Titanium has several favourable properties which include low density of 4.5 g/cm & is relatively high flexure strength comparable to that of cast forms of cobalt & stainless steel alloys. Titanium is very resistant to corrosion as a result it can be passivated by thin layer of titanium oxide which is formed instantly on its surface. This metal has the ability to form an oxide layer of nanometre thickness within a millisecond, & this oxide reforms if lost because of mechanical removal. If left unchecked this oxide layer can become thicker over time. Pure Titanium has the ability to form several oxides, including TiO, TiO_2_, & Ti_2_O_3_ of these, TiO_2_ is considered the most stable & is found after exposure to physiological conditions (5).

With the exception of pure titanium, the modulus of elasticity of Ti-6Al-4V is closer to that of bone than that of any other widely used metallic implant biomaterial. Newer titanium alloys have been developed, including Ti-13Nb-13Zr & Ti-15Mo-2.8Nb. These alloys utilize the other phase stabilizes instead of aluminium & vanadium and they may exhibit greater strength & corrosion resistance (5). The yield strength of Ti-6Al-4V Extra low interstitial (ELI) grade & Ti-6Al-4V alloys is 795 MPa & 860 MPa respectively is 68% & 78% greater than that of CPTi. Ti alloys are able to maintain the fine balance between sufficient strength to resist fracture under occlusal forces and to retain a lower modulus of elasticity for more uniform stress distribution across the bone implant interface (5).

Titanium is considered to be a biocompatible material. However, in light of recent clinical reports regarding contact dermatitis or granulomatous reactions to titanium upon its use in pacemakers, hip prostheses, surgical clips, or osteosynthesis, there is debate with regard to titanium allergies. In addition, titanium has also been reported to possibly cause generalized health problems, and the potential for adverse human tissue responses to titanium dioxide, which always covers the surface of titanium materials, has been reported. Chronic low-level metal exposure may occasionally result in metal sensitization and undesirable side effects. Dental implants, which were purported by the manufacturer to be made of ASTM grade I high-purity titanium (99.64%), the clinical report presents a suspected association of an allergic reaction with titanium dental implants. It appears that in rare circumstances, for some patients, the titanium used in dental implants may induce an allergic reaction (14).

## CERAMICS

Implant research has focused on discovering tooth colored implant material that improves the aesthetic appearance of dental implants and at the same time is highly biocompatible and able to withstand the forces present in oral cavity. Ceramic implants can withstand only relative low tensile or shear stress induced by occusal loads, but they can tolerate quite high levels of compressive stress. Ceramics can either be plasma sprayed or coated on to the metallic surfaces which can be more thermodynamically stable, hydrophilic, and non conductive of heat and electricity, thereby producing a high strength integration with bone (5). Aluminium oxide (Al_2_O_3_) is used as a standard biomaterial for ceramic implants because of its inertness (Biostability) with no evidence of adverse in vivo reactions. Zirconia (ZrO_2_) has also demonstrated a high degree of inertness, although alumina has higher surface wettability compared with other surfaces, such as those of metallic implants. These types of ceramic implants are not bioactive in that they do not promote the formation of bone (5).

Aluminium oxide (Al_2_O_3_) dental implants osseointegrated well but was withdrawn from market because of its poor survival rate. The strength and toughness of zirconia can be accounted for by its toughening mechanism, such as crack deflection, zone shielding, contact shielding & crack bridging. Prevention of crack propagation is of critical importance in high fatigue situations such as those encountered in mastication & parafunction. This combination of favorable mechanical properties makes zirconia a unique and stable material for use in high load situations (15). The biaxial flexural strength of zirconia implant ranges between 900 & 1100 MPa, while the weibull modulus ranges from 10-13. Surface treatment such as airborne particle abrasion and hand grinding have been found to improve flexural strength of zirconia implant. The uniaxial strength of zirconia has been found to be 409-899 MPa. Along with the strength, the fracture toughness is one of the first parameters to evaluate performance of a dental ceramic. Fracture toughness for zirconia implant has been found to be 4-6.2 MPa; also the stress distribution for yttrium-partially stabilized zirconia was similar to that of titanium (15). Surface topography and mechanical & chemical treatment of zirconia affect the shear bond strength of zirconia implants. Chemical treatment such as silanation of silicoated zirconia & application of zirconia has been found to increase the shear bond strength significantly.

Performance of Calcium phosphates as grafting and bone augmentation material is associated with the fact that bone is composed of 60-70% calcium phosphate. These materials are nonimmunogenic and biocompatible with host tissues. The two most commonly used calcium phosphates are Hydroxyapatite (HA) and tricalcium phosphate which is used as bone graft material to serve as template for new bone formation, because these materials promote and achieve direct bond of implant to hard tissues. Both also promote vertically directed bone growth as well as relatively strong bond to bone. Studies have shown that after 4 weeks of implantation, osteocytes accumulate adjacent to HA granules, indicating possibility of osteogenesis with implants. Use of these calcium phosphates as coating biomaterials for metallic implants is directly related to their physical & chemical properties. The more crystalline the HA coating the more resistant they are to clinical dissolution. Heat treatment after deposition process improves the crystallinity of HA .The amount of bone integration has been compared between metallic implants & ceramic coated implants in numerous studies, which suggests that there is a greater bone to implant integration with HA coated implants .However, other studies indicate there is no significant difference between coated & uncoated implants after months of integration, which implies early integration may be quite different (5).

Bioglass (SiO_2_-CaO-Na_2_O-P_2_O_5_-MgO) is another ceramic biomaterial which is classified as Bioactive as it stimulate the formation of bone. Despite their favorable osteo inductive ability they are also very brittle which makes them unsuitable for use of some stress bearing implant application. These materials are known to form a carbonated hydroxyapatite layer in vivo as a result of their calcium and phosphorus content. The formation of this layer is initiated by migration of calcium, phosphate, sodium and silica ions into the tissue as result of external pH changes. As elements are released silica rich layer and calcium –phosphorus layer is formed on the surface that stimulates osteoblasts to proliferate. These osteoblasts produce collagen fibrils that become incorporated into calcium phosphorus layer which forms a strong bone -bioglass interface which is about 100 to 200 µm thick. More often they are used as grafting materials for ridge augmentation or bony defects than as a coating material for metallic implant because the interface (5).

Another important parameter to be considered in the selection of an implant material is its affinity towards bacteria & plaque. Lesser plaque accumulation has been reported with zirconia implants. Bacteria such as S sanguis, Porphyromonas gingivalis, short rods, and cocci have shown lesser adherence to zirconia than to titanium surface. The adhesion of Streptococcus to zirconia has also been shown to similar to that to glass ceramics. There seems to be no difference between polished and glazed zirconia as far as adherence of bacteria is concerned (15).

## OTHER IMPLANT BIOMATERIALS

Titanium Foam - New Implant Surface August 8th, 2008 Canadian researchers at the NRC Industrial Materials Institute have developed a porous titanium foam implant said to mimic a metallic version of bone. The titanium foam is made by mixing titanium powder with a polymer, and then adding foaming agents that expand the polymer when heated. Later, through a high-temperature heat treatment, the polymer is removed and the titanium particles are consolidated to provide mechanical strength to the porous structure. The rough surface creates friction between the implant and the bone, and also allows bone growth into the pores to help fix the implant in place. Among its potential benefits, titanium foam could make dental implants less invasive (16).

A new material for dental implants with the brand name “Roxolid” was developed to overcome the mechanical limitations of pure titanium. The new material is composed of the two elements titanium and zirconium. This binary TiZr alloy has a significantly increased mechanical stability compared to titanium grade 4 with respect to elongation. The TiZr implants are manufactured with the SLActive surface like the titanium SLActive implants: Sand blasted, acid etched and then stored in 0.9% NaCl solution in order to maintain the clean oxide layer with its hydrophilic properties (17).

Polymers have lower strengths and lower elastic moduli and higher elongations to fracture compared with other classes of biomaterials they are thermal and electrical insulators and are relatively resistant to biodegradation. When compared with bone they have lower elastic moduli with magnitudes closer to soft tissues. Polymers have been fabricated in porus and solid forms for tissue attachment, replacement and augmentation and as coatings for force transfer to soft tissue and hard tissue regions. In general, polymers and composites of polymers are especially sensitive to sterilization and handling techniques. If intended for implant use, most cannot be sterilized by steam or ethylene oxide. More polymeric biomaterials have electrostatic surface properties and tend to gather dust or other particulate if exposed to semi clean oral environments (18).

Vitreous Carbon elicits a very minimal response from host tissues, is one of the most common implant biomaterial. Compared with the metallic implants, carbon is more inert under physiological conditions & has the modulus of elasticity similar to that of dentine and bone .Thus, carbon deforms at the rate similar to those tissues, enhancing the transmission of biomechanical forces. However carbon is susceptible to fracture under tensile and stress condition because of its brittleness and susceptibility to fracture under tensile stress in the presence of surface flaws, which is usually generated as component of flexural loading (5). Vitreous carbon is a 99.99 % pure form of carbon with a compressive strength of 50,000 to 1,00,000 pounds per square inch, a transverse strength of 10,000 to 30,000 psi and a modulus of elasticity between 3 -4 x 10^6^ psi. This modulus is similar to that of dentin; this is a significant factor in reducing shearing forces at the implant bone interface. This implant is formed by molding resin into the implant shape, heat treating it under nitrogen and then vacumizing it to evaporate the nitrogen, oxygen, hydrogen and any impurities included in the resin (19).

Pyrolytic carbon or LTI (low temperature isotropic carbon) are formed in a fluidized bed by the pyrolysis of a gaseous hydrocarbon depositing carbon onto a preformed substrate such as polycrystalline graphite. The silicon variety of pyrolytic carbon is prepared by code positing silicon with carbon to produce stronger implant material. The strength and its ability to absorb energy on impact is nearly 4 times greater than that of glassy or vitreous carbon. The modulus of elasticity of all isotropic carbon materials is almost similar to that of bone (20).

## SELECTING AN IMPLANT BIOMATERIAL

The American dental association outlines some acceptance guidelines for dental implants:

1. Evaluation of physical properties that ensure sufficient strength;
2. Demonstration of ease of fabrication and sterilization potential without material degradation;
3. Safety & biocompatibility evaluation, including cytotoxicity testing & tissue interference characteristics;
4. Freedom from defects;
5. At least two independent longitudinal prospective clinical studies demonstrating efficacy.

The surface properties of an implant are fundamental to near term and long term success of the device. Although carefully optimized surfaces cannot prevent implant failure due to mechanical instabilities, they can significantly improve the success rate of most types of implants. Wennerberg and co-worker have classified implant surface as Minimally rough (0.5-1 µm), Intermediately rough (1-2 µm), Rough (2-3 µm), Based on texture obtained Concave texture (mainly by additive treatments like HA coating and titanium plasma spraying) Convex texture (mainly by subtractive treatment like etching and blasting), Based on orientation of irregularities Isotopic surfaces which has the same topography independent of measuring direction & Anisotropic surfaces which has clear directionality and differ considerably in roughness (21).

Sandblasting a metal core with gritting agent creates modified surface. Aim to increase the irregularity of the surface using agents like alumina and titanium dioxide. Studies have shown that sandblasted samples showed largest variability in surface appearance. Sandblasting had been shown in some studies to allow adhesion, proliferation and differentiation of osteoblasts. On the other hand fibroblast were found to adhere with more difficulty to this surface; this could limit the soft tissue proliferation and potentially benefit bone formation(21).

Acid etched surface was proposed to modify the implant surface without leaving the residues found after the sandblasting process to avoid the non uniform treatment of the surface and to control the loss of metallic substance from the body of the implant. This is performed using hydrochloric, sulfuric acid, HF, and nitric acid in different combinations. The roughness before etching, the acid mixture, the bath temperature, and etching time all affect the acid etching process (3).

In the 1990s the study of a modified surface resultant from blasting followed by acid etching showed promising results.Surface is produced by a large grit 250-500 µm blasting process followed by etching with hydrochloric/sulfuric acid. The main objective is sandblasting results in surface roughness and acid etching leads to micro texture and cleaning. The resultant surface was constituted by uniformly scattered gaps and holes and it appeared to be slightly less rough than plasma sprayed surface which presented a deeply irregular texture that provided a less favorable environment for cell spreading. Authors conclude that the rate and degree of osseointegration is superior in sandblasted and acid etched implants they tend to promote greater osseous contact at earlier time points compared with plasma sprayed, coated implants and also present better osteoconductive properties and higher capability to induce cell proliferation than plasma sprayed surfaces (21).

Plasma sprayed implants are prepared by spraying molten metal on the titanium base which result in a surface with irregularly sized and shaped valleys, pores and crevices increasing the microscopic area by 6 to 10 times. This topography may improve the fixation of the implants by the growth of the bone into the coating forming a mechanical interlock.Plasma sprayed surfaces obtained with more reactive materials have been proposed to accelerate and enhance the bone growth into pores of the implant surface. TITANIUM PLASMA SPRAY (TPS) surface has been reported to increase the surface area of the bone implant interface and acts similarly to three dimensional surface which may stimulate adhesion osteogenesis. The surface area increase has been reported to be great as 600%. Porous surface in the range of TPS (150 to 400 microns) also increases the tensile strength of bone implant interface, resist shear forces and improve load transfer. An alkali modification was proposed after plasma spraying using sodium hydroxide solutions at 40 C for 24 hours. Authors suggest that alkali modification may be beneficial to reduce clinical healing times and thus to improve implant success rates (3).

Hydroxypaptetite (HA) coatings have a similar roughness and increase in functional surface area as TPS. A direct bone bond shown with HA coating and the strength of the HA to bone interface is greater than titanium to bone and even greater than TPS to bone (3). HA Plasma spraying involves the heating of Hydroxyapatite by a plasma flame at a temp of approx 15000-20000K. Then HA is propelled on to the implant in an inert environment like argon to a thickness of about 50-200 µm. Degrees of roughness (7-24 μm) was produced by plasma spraying. Sputtering is a process whereby, in a vacuum chamber, atoms or molecules of a material are ejected from a target by bombardment of high energy ions. The dislodged particles are deposited on a substrate, placed in a vacuum chamber. There are various sputtering techniques. However an inherent disadvantage is deposition rate is very slow. The sputtering technique has been used to coat homogeneous thin films <1 μm thick with a high adhesion strength to the substrate. Latest method of coating HA on to an implant surface is Pulsed laser deposition (PLD) is a thin film deposition technique where a high power pulsed laser beam is focused inside a vacuum chamber to strike a target of the material that is to be deposited. This material is vaporized from the target (in a plasma plume) which deposits it as a thin film on a substrate This process can occur in ultra high vacuum or in the presence of a background gas, such as oxygen. (Nd: YAG laser beam of 355-nm wavelength is used) (21).

## SUMMARY & CONCLUSION

The biomaterials discipline has evolved significantly over the past decades, and synthetic biomaterials are now constituted, fabricated, and provided to health care professionals as mechanically and chemically clean devices that have a high predictability of success when used appropriately within the surgical disciplines. Surface characterization and working knowledge about how surface and bulk biomaterial properties interrelate to dental implant biocompatibility profiles represent an important area in implant-based reconstructive surgery. Because of the abundance of different implant biomaterial & implant system, it is important to know the indications for their use. Perhaps the most important consideration is the strength of the implant biomaterial and type of bone in which the implant will be placed. The other factors to consider are implant design, abutment choices, abutment availability, surface finish and biomechanical consideration of restorative treatments. Modern dentistry is beginning to understand, realize and utilize the benefits of biotechnology in health care. Study of material sciences along with the biomechanical sciences provides optimization of design and material concepts for surgical implants.
